# The Role of Adipokines in the Pathogenesis of Psoriasis

**DOI:** 10.3390/ijms24076390

**Published:** 2023-03-28

**Authors:** Kajetan Kiełbowski, Estera Bakinowska, Piotr Ostrowski, Bartłomiej Pala, Ewa Gromowska, Klaudia Gurazda, Paweł Dec, Andrzej Modrzejewski, Andrzej Pawlik

**Affiliations:** 1 Department of Physiology, Pomeranian Medical University, 70-111 Szczecin, Poland; 2 Plastic and Reconstructive Surgery Department, 109 Military Hospital, 71-422 Szczecin, Poland; 3 Department of Surgery, Pomeranian Medical University, 71-422 Szczecin, Poland

**Keywords:** psoriasis, adipokines, leptin, adiponectin, inflammation, cytokines

## Abstract

Psoriasis is a chronic and immune-mediated skin condition characterized by pro-inflammatory cytokines and keratinocyte hyperproliferation. Dendritic cells, T lymphocytes, and keratinocytes represent the main cell subtypes involved in the pathogenesis of psoriasis, while the interleukin-23 (IL-23)/IL-17 pathway enhances the disease progression. Human adipose tissue is an endocrine organ, which secretes multiple proteins, known as adipokines, such as adiponectin, leptin, visfatin, or resistin. Current evidence highlights the immunomodulatory roles of adipokines, which may contribute to the progression or suppression of psoriasis. A better understanding of the complexity of psoriasis pathophysiology linked with adipokines could result in developing novel diagnostic or therapeutic strategies. This review aims to present the pathogenesis of psoriasis and the roles of adipokines in this process.

## 1. Introduction

Psoriasis is a chronic, immune-mediated skin condition that affects around 2% of the population globally [1]. One of the most challenging aspects of psoriasis is its diverse presentation. Symptoms can vary from patient to patient, leading to difficulty in correctly diagnosing the condition. Furthermore, although diagnostic criteria are available to support a diagnosis, they do not always provide the best indication of severity or treatment. This means that some forms of psoriasis may be missed or mismanaged due to misdiagnosis. Age, gender, geography, and ethnicity contribute to the variability in psoriasis prevalence, probably due to genetic and environmental factors. In some African and Asian communities, it is less common, whereas it can reach up to 11% in Scandinavian populations [2]. Psoriasis is uncommon in some ethnic groups, such as the Japanese [3], and may not exist among Australian aborigines [4]. The disease often coexists with other conditions such as cardiovascular diseases, depressive illness, and psoriatic arthritis. Although the exact causes of psoriasis are poorly understood, there are several risk factors that have been identified, including familial history and environmental risk factors such as smoking and obesity [5].

Psoriasis is generally classified into several types, including plaque, guttate, inverse, and pustular. Plaque psoriasis is the most common type, accounting for approximately 80% of cases. It is characterized by raised, red patches on the skin covered with white or silver scales. Guttate psoriasis is the second most common type. It is characterized by small, water-drop-shaped lesions on the skin. Inverse psoriasis appears as red lesions in body folds such as the armpits or groin. Pustular psoriasis appears as white blisters filled with pus surrounded by red skin. Finally, psoriasis can be a psychologically and emotionally taxing condition to manage, with mood swings, stigma, and other mental health issues contributing to its overall negative impact on quality of life [6]. Costs are significant for both the healthcare systems and the patients [7]. Heart disease, depression, and psoriatic arthritis are all linked to psoriasis [8].

### 1.1. Pathogenesis of Psoriasis

The pathogenesis of psoriasis is incompletely understood, but it is believed to involve a combination of genetic and environmental factors. Pathologically, psoriasis is associated with an increase in the production of pro-inflammatory cytokines, such as tumor necrosis factor-alpha (TNF-α), interleukins (IL-1 and IL-17), and interferon-γ (IFN-γ), that drive the development of the disease. They cause epidermal keratinocyte hyperproliferation, differentiation defects, and the excessive production of pro-inflammatory mediators. Furthermore, psoriatic lesions are associated with an increased amount of dendritic cells and T cells [9]. Comorbidities of psoriasis, including metabolic syndrome, obesity, diabetes, and cardiovascular disease, have been attributed to the systemic inflammation caused by these cytokines. Other emerging implications of psoriasis pathogenesis involve impaired extracellular matrix deposition, altered Toll-like receptor expression, and aberrant antimicrobial activity. As we gain a more comprehensive understanding of the underlying inflammatory processes, clearer roles emerge for the potential therapeutic targets currently available to physicians treating individuals with chronic psoriasis [8,10].

Card14 mutations are important genetic risk factors for psoriasis. These mutations lead to the overexpression of pro-inflammatory cytokines, which causes chronic inflammation and leads to the development of psoriasis lesions. Stress has also been shown to trigger or worsen psoriasis flares, although the exact mechanism by which it does so is not fully understood. Various infections have also been linked to the development or exacerbation of psoriasis, including streptococcal throat infections [11,12], HIV infections [13], and hepatitis C infections [14]. Finally, exposure to certain irritants such as smoke is also associated with psoriasis development [15]. Therefore, psoriasis is a genetically predisposed, chronic inflammatory skin condition characterized by anomalies in the immune system, increased keratinocyte proliferation, poor epidermal cell differentiation, and increased angiogenesis [16].

### 1.2. Problems in the Diagnosis and Management of Psoriasis

As a disease that predominantly occurs in developed countries, psoriasis still poses a therapeutic and treatment challenge. Although recent advances in the understanding of the pathophysiology have shed light on this condition, some crucial problems in psoriasis diagnosis and management need to be addressed. One of the primary challenges of psoriasis diagnosis is reliably distinguishing it from other skin conditions that manifest similar symptoms, such as atopic dermatitis, seborrheic dermatitis, lichen planus, and eczematous contact dermatitis [10]. In usual circumstances, the morphology of the skin lesions and their distinctive locations are used to diagnose psoriasis (scalp, proximal surfaces of the elbows and knees, sacral region, nails). Taking a skin biopsy from the lesion and histologically verifying the diagnosis is advised if there is any uncertainty regarding the accuracy of the clinical diagnosis. In some circumstances, dermoscopy can be beneficial, particularly when treating nails and scalp psoriasis [17,18].

Once properly diagnosed, effective management requires individualized care based on their symptoms, lifestyle, and overall health. The available treatment options are numerous, from topical agents and phototherapy to biological therapies and systemic treatments. As there is no “one-size-fits-all” approach to managing psoriasis, choosing the best option for each patient can be difficult. Additionally, regular determination of the severity of the disease is very important to be able to choose the right treatment at the right time. The Psoriasis Area and Severity Index (PASI) and body surface area (BSA) are used to assess the severity of the disease and the extent of psoriasis, respectively. The degree and severity of erythema, thickness, and scaling within psoriatic lesions are evaluated by the PASI score. With a scale from 0 to 100, the BSA index shows what proportion of the body’s surface area is taken up by psoriatic lesions. The rule of nines, initially used to estimate burn area, is used for determining the BSA value [19]. The aim of treatment is to control symptoms and induce complete remission of skin changes. This objective is not always achievable, especially in patients with advanced illness. Therefore, if the PASI is reduced by at least 90% after treatment, it can be deemed successful (PASI-90) [20]. The treatment may also be considered successful if it lowers the DLQI (Dermatology Life Quality Index) to 5 points with the PASI reduced by at least 75%. A failure to see sufficient progress during treatment (PASI < 50 or PASI ≥ 50 and <75, with DLQI > 5 points) should be an indication for treatment modification [18,21].

### 1.3. Adipokines

Physiologically active proteins known as adipokines (or adipocytokines) are largely produced by adipocytes, whereas many of their components are also expressed and secreted by other cells [22]. Adipokines represent a functional category of various proteins and peptides involved in cell signaling, as opposed to protein families, which have a shared domain structure and a homologous, conserved amino acid sequence. In recent years, adipokines have gained increasing attention, particularly for their role in autoimmune processes. Adipokines such as leptin, adiponectin, and resistin are proteins that are released into the circulation by adipocytes and have been found to have profound immunomodulatory effects. These adipokines have been implicated in the regulation of both innate and adaptive immunity, making them essential components of healthy physiological function. Furthermore, their expression has been linked to several pathological states, including inflammatory and autoimmune diseases, obesity, and metabolic disease [23]. Overall, the human body has several hundred adipokines with a variety of biological characteristics. The most common classification separates pro- and anti-inflammatory adipokines and centers on their inflammatory qualities. A persistent, low-grade inflammatory state is created by the upregulation of pro-inflammatory adipokines, which also contributes to metabolic dysfunction. On the other hand, several anti-inflammatory adipokines are also secreted by adipose tissue, and their function in these processes is actively being researched [24]. Thus, understanding and harnessing the power of adipokines holds great potential in developing therapeutic strategies to treat these conditions.

Adipokines are known to play a role in inflammation and immune responses, both of which are involved in psoriasis. There are numerous studies examining the connection between psoriasis and the plasma or tissue expression levels of various adipokines, but precise mechanisms still need to be investigated. This may be due to methodological variations or incomparable study populations regarding the severity of the psoriasis and/or comorbidities [25]. For example, levels of leptin and resistin are elevated in obese individuals with psoriasis compared to those without the condition. Skin biopsy samples examined by immunohistochemistry for leptin and leptin receptor expression revealed an elevated expression in patients with severe psoriasis. Moreover, a significant association between the duration of the disease and serum leptin levels, tissue leptin, and tissue leptin receptor expression was demonstrated. Leptin could be used to measure the severity and recurrence of psoriasis [26,27]. According to another study, psoriasis patients who are obese have lower amounts of circulating adiponectin. For obese psoriasis patients, a strong negative connection to adiponectin was observed when compared to the pro-inflammatory cytokine IL-6 [28]. To fully comprehend the role of adipokines in psoriasis and other autoimmune diseases, more studies are required. Yet, these latest investigations imply that adipokines might be crucial in the emergence and advancement of these disorders. Adipokines may help the growth of psoriasis, although the precise method by which they do so is unclear. It is evident that they contribute significantly to the development of this illness, nevertheless.

## 2. Adiponectin

### 2.1. Structure, Signaling Pathways, and Physiological Role of Adiponectin

Adiponectin was first described by Scherer and colleagues in 1995. The freshly discovered protein was referred to as the adipocyte complement-related protein of 30 kDa (Acrp30) [29]. It was also termed AdipoQ [30], apM1 [31], and GBP28 [32]. Only a few years later, the first studies started to reveal the role of adiponectin in regulating blood glucose levels [33], which further confirmed that adipose tissue is an endocrine organ [34]. After years of research, it is now clear that adiponectin has a strong impact on glucose and lipid metabolism. Furthermore, it modulates inflammatory responses and has anti-atherogenic and anti-diabetic properties [35,36].

**Figure 1:**
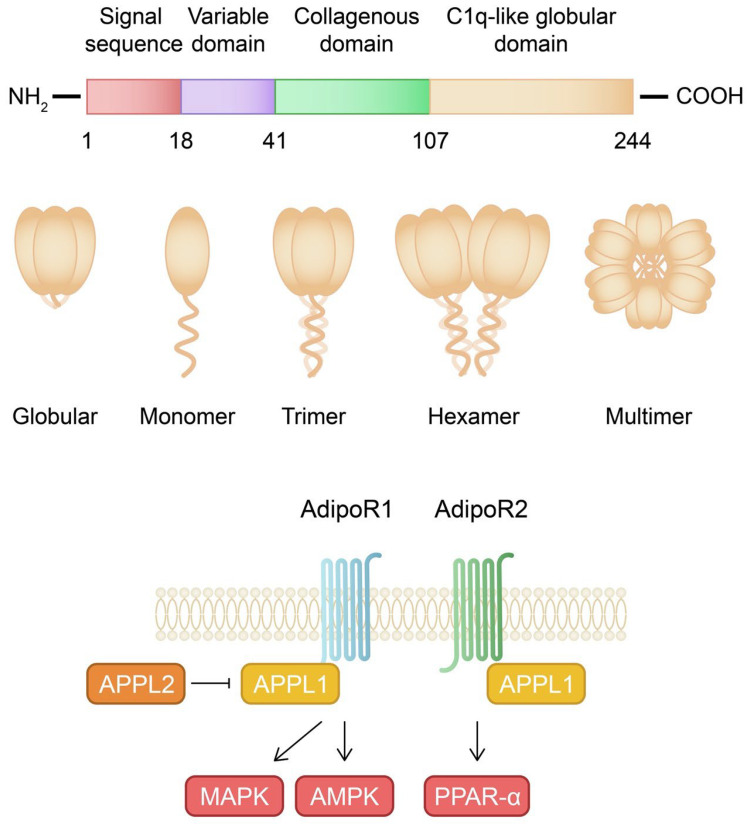
Schematic representation of the adiponectin isoforms and signaling. AdipoR1—adiponectin receptor 1; AdipoR2—adiponectin receptor 2; APPL—adaptor protein containing PH domain, PTB domain, and leucine zipper motif-1; AMPK—AMP-activated protein kinase; MAPK—mitogen-activated protein kinase; PPAR—peroxisome proliferator-activated receptor.

Human adiponectin protein contains 244 amino acids. It is composed of four regions, including a signal sequence at the N-terminus, a variable region, a collagenous domain, and a complement 1q-like globular domain at the C-terminus [37]. Adiponectin protein undergoes multimerization into trimers (low molecular weight, LMW), hexamers (medium molecular weight, MMW), and multimers (high molecular weight, HMW) (Figure 1) [38]. These isoforms differently regulate metabolism. HMW is considered a major isoform involved in glucose metabolism. Accordingly, adiponectin mutations responsible for diabetes and hypoadiponectinemia are associated with impaired multimerization [39,40]. The levels of HMW complexes are reduced in obese, insulin-resistant, as well as atherosclerotic patients [41,42]. A proper multimerization process requires a disulfide bond between the cysteine residues [40]. Furthermore, the modification of lysine in the collagenous domain [43] and the mutation of a conserved tryptophan residue in the N-terminus also disrupt HMW assembly [44]. In addition, testosterone and TNF-α reduce the level of HMW adiponectin [45,46]. Globular adiponectin represents another variant of the protein, which is generated through the proteolytic cleavage of the full-length adiponectin. Globular adiponectin is biologically active and promotes the accumulation of triglycerides in adipocytes [47,48].

Adiponectin binds to AdipoR1, AdipoR2, and T-cadherin. Furthermore, it has also been demonstrated that it can bind to calreticulin. However, the binding affinity depends on the isoform of the adiponectin protein [49,50,51]. The structure of AdipoR1/AdipoR2 involves an intracellular N-terminal region, seven transmembrane domains, and an extracellular C-terminal region. Therefore, the topology of these receptors differs from the G-protein coupled receptors [52]. The downstream signaling of adiponectin receptors includes AMP-activated protein kinase (AMPK) and peroxisome proliferator-activated receptor (PPAR)-α [53]. AMPK belongs to a major signaling pathway involved in insulin sensitivity by promoting glucose uptake (GLUT4 translocation) and glycolysis [54]. Moreover, it is considered that AMPK may also inhibit gluconeogenesis, but studies have found that this process can be AMPK-dependent and -independent [55,56,57,58,59]. The stimulation of PPAR-α is associated with an atheroprotective plasma lipid profile. It increases the levels of HDL and decreases the plasma levels of triglycerides. Furthermore, PPAR-α inhibits pro-inflammatory signaling pathways [60,61]. Additionally, the adiponectin receptor directly interacts with APPL1 (adaptor protein containing PH domain, PTB domain, and leucine zipper motif-1) [62]. In rat cardiomyocytes, APPL1 interacts with both receptors, but adiponectin elevated the interaction only with AdipoR1 [63]. APPL1 is thought to mediate the activation of AMPK through the cytosolic translocation of LKB1, an AMPK kinase [63,64,65]. In C2C12 myocytes, APPL1 was found to stimulate another major signaling pathway, the p38 mitogen-activated protein kinase (MAPK) pathway [49,66]. An interaction between APPL1 and AKT has also been identified [67,68]. However, this interaction has been associated with the insulin-signaling pathway, as adiponectin alone might not trigger the activation of Akt [62,69]. Therefore, APPL1 is involved in major cellular functions. Wen and colleagues demonstrated that APPL1 knockdown in 3T3-L1 preadipocytes disrupted differentiation into mature cells and inhibited autophagy [70]. APPL2, an isoform of APPL1, negatively impacts adiponectin signaling and competes with APPL1 in the interaction with AdipoR1 [71]. Adiponectin signaling is enhanced with the overexpression of heat shock protein 60, which stabilizes the adiponectin receptor [72]. The binding of adiponectin to T-cadherin has been correlated with protective effects in tubular renal injury [73], atherosclerosis [74], and heart [75], among others.

The role of adiponectin signaling has been extensively investigated in the function of immune cells. In human macrophages stimulated with lipopolysaccharide (LPS), adiponectin inhibits the expression of pro-inflammatory TNF-α and IL-6. The authors also showed that adiponectin induces the expression of genes encoding anti-inflammatory proteins, such as A20, SOCS3, and BCL3, among others [76]. In addition, Ohashi and colleagues demonstrated that adiponectin promotes the expression of anti-inflammatory phenotype (M2) markers such as CD163, IL-10, and mannose receptor in human monocyte-derived macrophages [77]. In contrast, a subsequent study revealed that the role of adiponectin in macrophage function is much more complex. Cheng and colleagues demonstrated that adiponectin induces the expression of genes encoding both M1 and M2 phenotype proteins, favoring the M1 pro-inflammatory phenotype. The authors suggest that the induction of pro-inflammatory pathways makes macrophages resistant to further inflammatory stimuli [78]. Interestingly, the M1 phenotype might be associated with reduced expression of AdipoR1 and AdipoR2 compared to the M2 subtype. Additionally, adiponectin enhances pro-inflammatory cytokine secretion in the M1 macrophage population (TNF-α, IL-6), whereas it induces anti-inflammatory secretion in M2 cells (IL-10) [79]. Therefore, adiponectin may promote both pro- and anti-inflammatory signaling, which may depend on the variant of macrophages. A study by Jin and Wang demonstrated that the treatment of mouse Raw 264.7 cells for 3 h with globular adiponectin promoted the expression of pro-inflammatory cytokines, such as TNF-α, IL-6, and monocyte chemoattractant protein (MCP-1). These effects are thought to occur through the translocation of NF-κB to the nucleus. However, these results might not be dependent on AdipoR1 or AdipoR2. In contrast, full-length adiponectin promoted macrophage migration but did not affect pro-inflammatory cytokine production [80]. Intriguingly, Haugen et al. showed that immunomodulatory effects may result from the adiponectin isoform. The authors found that HMW and globular variants promoted NF-κB activity [81].

Despite macrophages, adiponectin plays a role in the functioning of other immune cells, such as T cells. It has been found that T cells express most adiponectin receptors intracellularly. The stimulation of T cells with antigens promotes the expression of AdipoRs on the cell surface. Adiponectin decreases the number of antigen-specific T cells and suppresses the production of IFN-γ, IL-2, and TNF-α [82]. Furthermore, Surendar and colleagues found that the stimulation of naïve CD4+ cells with adiponectin leads to a reduced number of IFN-γ+ T cells [83]. Similarly, a separate study showed that adiponectin suppresses the ox-LDL-induced differentiation of Th1 and Th17 cells [84]. Additionally, in line with previous studies, Zhang et al. found that adiponectin suppressed the Th1 and Th17 cytokines IFN-γ and IL-6, respectively [85]. Nevertheless, the role of adiponectin on T cells might depend on cellular context (e.g., the effect of various patterns of antigens or cytokines), as some reports showed that adiponectin may promote IFN-γ T cells [78,86].

### 2.2. Impact of Adiponectin on Skin and Joints

Adiponectin protein is a significant regulator of metabolism and has a range of immunomodulatory properties. However, recent evidence also points to important modulatory roles of adiponectin signaling on the skin and joint tissues. To begin with, Sun and colleagues evaluated whether AdipoRon, an agonist of AdipoRs, may impact skin inflammation. The authors found that AdipoRon dose-dependently reduced the expression of IL-1β, IL-6, and TNF-α in mouse skin samples. Furthermore, adiponectin agonist suppressed apoptosis [87]. Moreover, Tu et al. demonstrated that peritoneal adiponectin injections in rats with skin flaps resulted in higher survival areas, higher levels of vascular endothelial growth factor, denser microcirculation, and increased activity of superoxide dismutase [88]. Recent studies highlight the important role of adiponectin in regulating skin fibrosis, as adiponectin-knockdown mice develop more fibrosis after exposure to pro-fibrotic conditions compared with wild-type models [89]. Furthermore, a negative correlation between mRNA adiponectin expression and modified Rodnan skin score (MRSS), which measures skin thickness, was demonstrated in patients with systemic sclerosis [90]. On the other hand, a study by Masui et al. showed a positive correlation between serum adiponectin and total skin thickness score. Nevertheless, diffuse cutaneous systemic sclerosis was associated with a lower serum adiponectin level compared with the limited subtype of the disease. Additionally, the authors found a positive correlation between the serum adiponectin level and the duration of the disease. Therefore, a reduction in the serum protein level may contribute to the early development of the disease and the initiation of skin fibrosis but is unrelated to disease progression [91]. In addition, adiponectin protein reduces collagen expression in fibroblasts with and without pre-treatment with lipopolysaccharide [92]. Therefore, these studies point to the important role of adiponectin in preserving inflammation quiescence and preventing fibrosis.

In addition, adiponectin modulates the function of another important skin cell population, keratinocytes. To begin with, human keratinocytes express adiponectin receptors [93]. Secondly, it can reduce the elevated expression of human beta-defensin 2 (hBD2) in UV-treated keratinocytes. The overexpression of hBD2 has been previously correlated with elevated keratinocyte proliferation [94]. Furthermore, adiponectin reduces hBD2 levels stimulated by H_2_O_2_, but cells stimulated with this adipokine alone also show elevated production of antimicrobial peptides [95]. Kawai and colleagues demonstrated that adiponectin has a pro-apoptotic effect on keratinocytes [96]. Furthermore, through MAPK signaling, activator protein 1, and silent mating-type information regulation 2 homolog (SIRT1), adiponectin promotes the expression of filaggrin in keratinocytes, which is a key element in preserving skin barrier function [97,98].

Adiponectin has also been found to impact joint tissues and may take part in the pathogenesis of osteoarthritis (OA) and rheumatic arthritis (RA). However, taking its anti-inflammatory roles described previously, its contribution to joint diseases is thought to be controversial. For instance, the expression of AdipoR seems to be more abundant in lesional areas of OA cartilage. Furthermore, the protein induces the expression of pro-inflammatory cytokines, such as IL-6 and MCP-1, together with metalloproteinases involved in the degradation of cartilage [99,100]. The production of MMP-3 by human chondrocytes is thought to be mediated by AdipoR1, AMPK, p38, and NF-kB [101]. Furthermore, a positive association has been identified between serum adiponectin levels and radiographic knee OA severity [102]. Moreover, the synovial fluid concentration of adiponectin positively correlates with markers of aggrecan degradation [103]. In RA synoviocytes, stimulation with adiponectin promotes the production of IL-8, IL-6, MMP-13, MMP-1, and VEGF [104]. Accordingly, Wang et al. demonstrated that the inhibition of AdipoR1 decreases RANKL expression and prevents joint tissue damage in collagen-induced arthritic mice [105]. Interestingly, these observations might result from the local adiponectin effects. Ebina and colleagues demonstrated that systemic adiponectin prevents joint chemokine deposition and decreases tissue degradation [106]. Therefore, these studies suggest that adiponectin acts locally on joint tissues, which results in a pro-inflammatory environment and the promotion of joint diseases.

### 2.3. The Role of Adiponectin in Psoriasis

**Figure 2:**
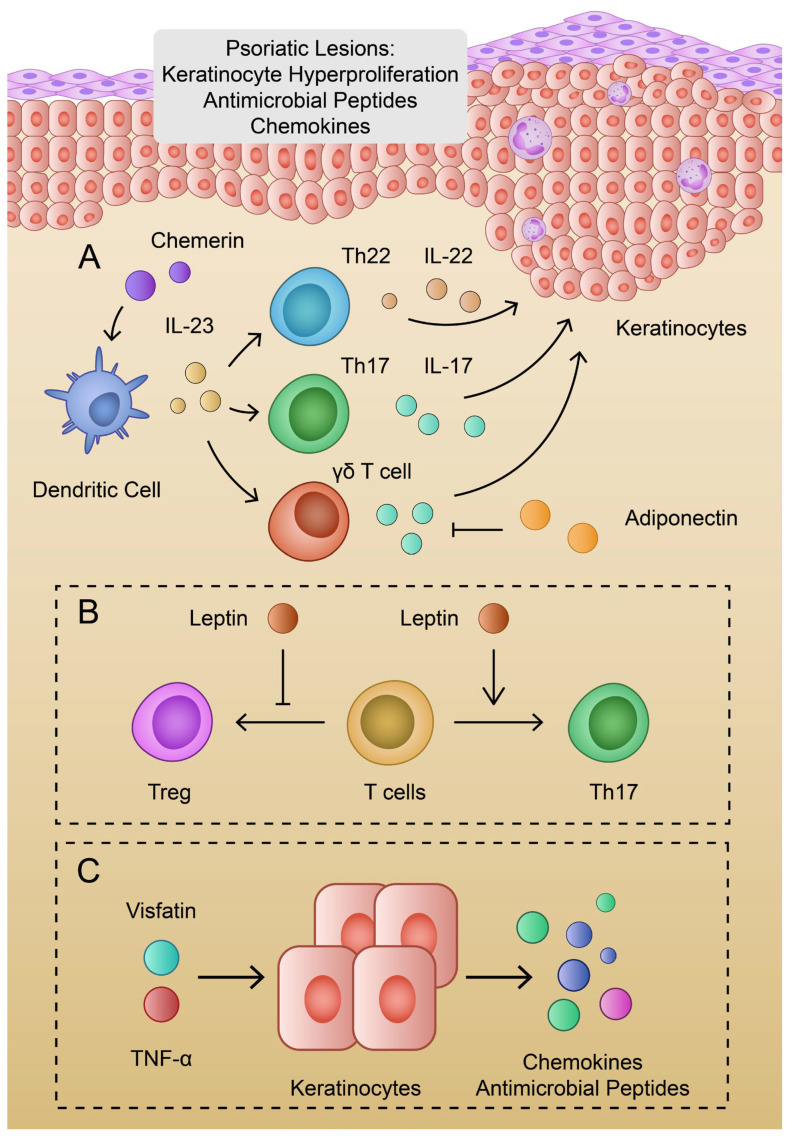
(**A**): Schematic representation of the pathogenesis of psoriasis and the roles of chemerin and adiponectin; (**B**): Impact of leptin on T cell variants; (**C**): Visfatin and TNF-α promote production of chemokines and antimicrobial peptides.

Psoriasis, a chronic inflammatory skin disease, is associated with dysfunctional differentiation and hyperproliferation of keratinocytes. Skin lesions are characterized by the infiltration of immune cells, together with neovascularization. Separate variants of the disease are characterized by different inflammatory patterns, such as TNF-α, IL-23, and Th17 or IL-36α, IL-36γ, and IL-1β [16]. Van der Fits et al. proved that IL-23 and IL-17 are key cytokines involved in the pathogenesis of psoriasis, as IL-23p19- and IL-17RA-deficient mice demonstrated suppressed psoriatic lesions compared to wild-type models [107]. IL-23 is mainly secreted by dermal dendritic cells and macrophages. It promotes the production of further cytokines by Th17 cells, such as IL-17, and IL-22 [108,109]. In contrast, IL-17 is largely produced by T cells in psoriatic lesions, whereas keratinocytes are considered as the main target cells. Subsequently, IL-17 promotes chemokine secretion and the production of other cytokines responsible for the promotion of skin inflammation and psoriatic changes [110]. IL-23-stimulated γδ-T cells are another source of IL-17 in psoriasis [111]. The elevation of γδ-T cells is observed in psoriatic and recurrent lesions [112]. Additionally, IL-22 is largely produced by Th22 cells, which are elevated in psoriatic patients [113,114,115]. IL-6 and IL-23 facilitate the development of Th22 cells [116] (Figure 2). T cells expressing CD4 and Forkhead box protein 3 (Foxp3) are referred to as regulatory T cells (Tregs). The dysregulation of Tregs has been identified in the pathogenesis of autoimmune diseases and cancer. These cells are capable of secreting anti-inflammatory cytokines, such as IL-10 [117]. The dysregulation of the Th17/Treg balance has been identified in psoriasis. To begin with, etanercept (anti-TNF-α agent) has been found to alleviate psoriasis in a mice model and inhibit pro-inflammatory cytokines. Psoriasis was associated with elevated Th17 and reduced Tregs, and etanercept has been found to normalize these cell populations [118]. Secondly, Shi et al. showed that the expression of IL-21 and IL-21R in CD4+ cells is elevated in psoriatic skin lesions. Moreover, the authors demonstrated that IL-21 promotes Th17 and disrupts Treg differentiation [119]. Therefore, several T cell subtypes play roles in the pathogenesis of psoriasis [120]. Interestingly, IL-23 was also found to stimulate the polarization of macrophages into different variants than M1 or M2. IL-23-treated macrophages were found to produce IL-17, IFN-γ, and IL-22 [121].

Recent evidence highlights the important and potentially beneficial role of adiponectin in psoriasis. To begin with, patients with psoriasis have a significantly decreased level of adiponectin compared to healthy controls [122,123]. Secondly, the treatment of psoriasis leads to an elevation of the serum adiponectin level [124]. In addition, the expression of adipoQ is reduced in psoriatic skin lesions when compared to healthy skin [125]. Adiponectin may be correlated with IL-23 and IL-17. Kochumon and colleagues found negative correlations between IL-23 gene expression and the level of adiponectin in patients with high levels of LDL cholesterol [126]. Furthermore, Shibata et al. used an adiponectin knockdown and wild-type mice to evaluate the expression of psoriasis cytokines. The authors found that a deficiency of adiponectin further promotes the expression of IL-23p19 and IL-17 in skin treated with imiquimod. Interestingly, intraperitoneal injection with adiponectin resulted in the inhibition of IL-17 production in adiponectin-deficient mice. Moreover, in an in vitro analysis, adiponectin suppressed the production of IL-17 from IL-23-stimulated dermal γδ-T cells [127]. In addition, an adiponectin-derived peptide, P5, which acts through the AdipoR1 receptor, was found to inhibit IL-17A mRNA expression in γδ-T cells and alleviate imiquimod-induced psoriasis in mice [128,129]. Furthermore, the previously mentioned hBD2 is one of the specific markers of psoriasis, which is also involved in a broad range of psoriasis-specific inflammatory pathways. It has been previously suggested to monitor hBD2 throughout the treatment process [130]. HBD2 expression is correlated with several cytokines, including IL-23. Kanda et al. demonstrated that IL-23 potentiated the IL-1β-induced production of hBD2 in keratinocytes [131]. Therefore, adiponectin might serve a protective role in psoriasis through a negative correlation with IL-23 and hBD2. Moreover, adiponectin might impact the Tregs population. Co-culture of CD4+ T cells with dendritic cells conditioned with adiponectin leads to the elevated ratio of Treg cells [132]. Furthermore, Ramos-Ramirez and colleagues revealed that globular adiponectin and AdipoRon can promote the expression of Foxp3 and promote the secretion of IL-10 [133].

Adiponectin acts through the AMPK signaling pathway. Interestingly, the activity of AMPK has been correlated with the modulation of psoriasis, which might indirectly suggest the effects of adiponectin. The expression of AMPK and its phosphorylated form are decreased in the skin of patients with psoriasis [134]. Shen et al. used a mouse model of psoriasis to evaluate the role of the AMPK signaling agonist in the development of psoriasis. The authors found that the use of a signaling agonist was associated with decreased skin thickness, whereas the AMPK inhibitor promoted disease severity [135]. In line with these findings, Garcin and colleagues demonstrated that AMPK inhibitor leads to hyperkeratosis and the promotion of IL-20 [136]. However, conflicting data exist about AMPK phosphorylation by adiponectin in keratinocytes [93,95]. Moreover, it is worth mentioning the sirtuin 1 (SIRT1) pathway, which is activated by AMPK and is suppressed in imiquimod-stimulated mouse skin [137,138,139]. Hong and colleagues demonstrated that treating keratinocytes with adiponectin promotes SIRT1 expression [140]. Furthermore, SIRT1 negatively regulates the signal transducer and activator of transcription 3 (STAT3), which is a mediator of IL-22 signaling and a member of the leptin signaling pathway [141].

## 3. Leptin

### 3.1. Leptin in Physiology and Pathology

**Figure 3:**
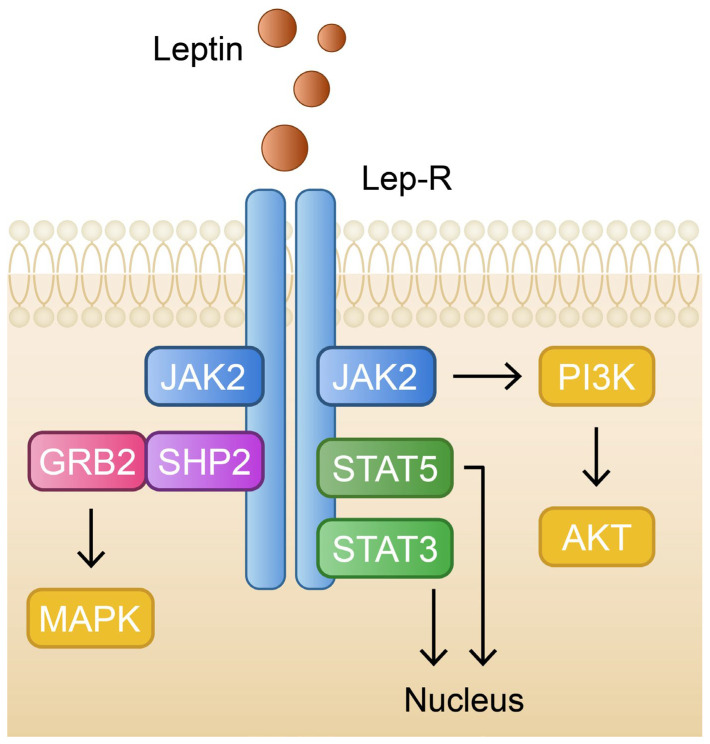
Schematic and simplified representation of the leptin signaling pathway. GRB2—growth factor receptor-bound protein 2; JAK2—janus kinase 2; MAPK—mitogen-activated protein kinase; PI3K—phosphoinositide 3-kinase; PIP2—phosphatidylinositol 4,5-biphosphate; SHP2—Src homology-2 domain-containing protein tyrosine phosphatase-2; STAT3—signal transducer and activator of transcription 3; STAT5—signal transducer and activator of transcription 5.

Leptin is one of the most relevant protein hormones, produced mainly by adipocytes in our white adipose tissue [142]. Its paramount function is a reduction in the sense of hunger by acting on the leptin receptors in the hypothalamus. Leptin concentration in blood is positively correlated with the amount of adipose tissue, hence increased leptin levels are encountered in people with obesity [143]. The presence of a leptin receptor was also identified in the basal layer and the hair follicle papilla cells in the epidermis. Stimulating them, leptin can activate JAK2 kinase which induces the tyrosine phosphorylation of the STAT3, SHP2, and PI3K proteins. Activated and dimerized STAT3 migrates to the nucleus, where it causes the expression of genes such as the suppressor of cytokine signaling 3 (SOCS3) [144,145]. All these pathways lead to mitochondrial metabolic activation and the efficiency of energy utilization. As a consequence, cellular proliferation and differentiation, as well as the modulation of angiogenesis, are stimulated in the epidermis layer [146]. Thus, the malfunction of any of the aforementioned signaling pathways can be associated with impaired wound healing or pathogenesis of skin diseases. Suppressing STAT3 is associated with the alleviation of keratinocyte inflammation [147]. Moreover, leptin is described as a hormone with pleiotropic effects due to its impact on hematopoiesis, thermogenesis, bone metabolism, the regulation of sexual reproduction, as well as immune homeostasis [148] (Figure 3).

Leptin’s influence on the immune system seems to be a significant factor in the pathogenesis of autoimmune disorders. During systemic inflammation, cytokines such as TNF-α, IL-6, and IL-1β can trigger adipocytes to upregulate leptin synthesis and expression with potentially disastrous effects. For instance, glomerulosclerosis may develop by renal glomerular endothelial cells over proliferation and upregulation of TGF-β expression, which triggers the deposition of extracellular matrix material in the glomerulus and subsequently can lead to proteinuria [149]. Leptin overproduction can also stimulate the recruitment and migration of monocytes to the intima of blood vessels and consequently increase the secretion of atherogenic cytokines, resulting in atherosclerosis [150]. In a state of chronically increased leptin blood concentration among people with obesity, certain tissues may develop leptin resistance, which can contribute to fat accumulation in the liver. The upregulated synthesis of free fatty acids may induce liver inflammation and fibrosis, mainly by the peroxidation of accumulated lipids, as well as the overproduction of reactive oxygen species (ROS) [151]. Liver tissue fibrosis is then promoted by hepatic stellate cells (HSCs) activated by procollagen I, TGF-B1, and smooth muscle actin produced in the aforementioned pathways. Furthermore, leptin appears to be a probable mitogen for HSCs and, at the same time, an inhibitor of HSCs’ apoptosis process by affecting the Akt-dependent pathway and extracellular signal-regulated kinase (ERK) [152].

### 3.2. The Role of Leptin in Psoriasis

According to the data available in the current literature, leptin might contribute to the development of psoriasis. Firstly, serum leptin and the expression of its receptor are elevated in severe psoriasis compared to mild disease and controls [26]. This finding was also confirmed in the meta-analysis by Zhu et al. when comparing patients with controls [153]. It enhances the secretion of various cytokines such as IL-1, IL-6, TNF-α, and CXCL8. Consequently, these mediators stimulate Th1/Th17 to overproduce IL-17 and IL-23 that are highly implicated in the pathogenesis of psoriatic arthritis [122,146]. Furthermore, several studies demonstrated that leptin promotes the differentiation of Th17 cells [154,155]. Yu et al. reported that leptin-deficient splenocytes stimulated with recombinant leptin resulted in an elevation of IL-17+ cells. Furthermore, the authors found that leptin increases the expression of the retinoic acid receptor-related orphan nuclear hormone receptor family (RORγt), which is associated with Th17 differentiation [156]. Leptin can also enhance granulocytes’ chemokinesis to the psoriatic skin and delay their apoptosis at this location. Activated neutrophiles might be another source of IL-17 excess. As a result of cytokine storm, psoriatic-related genes are overexpressed and cornified cell maturation is impaired [157]. Human keratinocytes treated with IL-17A and leptin demonstrate elevated gene expression of chemokines (*CXCL8*, *CXCL1*, *CCL20*) [158]. Leptin also potentiates hBD2 secretion in IL-1β-treated keratinocytes through the MAPK and JAK2 pathways [159]. In addition, leptin is associated with an impaired Tregs population. De Rosa and colleagues demonstrated that neutralizing leptin monoclonal antibodies enhances the proliferation of Tregs [160]. Furthermore, leptin decreases IL-10 production from CD4+ T cells from patients with asthma [161]. In line with previous findings, Wang et al. showed that leptin receptor antagonist promotes Foxp3 and inhibits IL-17 in the thyroid gland of a mouse model with experimental autoimmune thyroiditis [162]. Interestingly, Tregs from psoriasis patients are prone to differentiate into IL-17-secreting cells [163]. Moreover, the PASI score seems to be proportionate to the actual leptin concentration in serum [26]. However, this finding was not observed in another study regarding leptin plasma levels [122]. 

## 4. Other Adipokines and Their Role in Psoriasis

### 4.1. Visfatin

Visfatin is an adipokine identified in 2004 [164] and named after the suggestion that it is produced and excreted primarily in visceral fat. Visfatin is highly conserved throughout animal evolution. It has a molecular weight of 52 kDa and its gene encodes 491 amino acids. It is identical to pre-B cell colony-enhancing factor (PBEF), described in 1994 as a cytokine produced by lymphocytes, acting on lymphocyte maturation and inflammatory regulation. Visfatin is produced not only in human leukocytes and adipose tissue but also in human and animal liver and muscle cells [165], animal adipocytes, and kidney and heart [166]. Visfatin was found to be released primarily by macrophages rather than adipocytes in visceral adipose tissue. In this regard, there is ample evidence to suggest that visfatin is expressed by macrophages infiltrating adipose tissue and produced in response to inflammatory signals [167]. Visfatin has pleiotropic effects on various cells. For instance, it can promote the production of VEGF and MMP and interact with MAPK and phosphatidylinositol 3-kinase/protein kinase B (PI3K) signaling pathways, which leads to increased angiogenesis [168]. In addition, visfatin plays a regulatory role in cell proliferation and apoptosis [169]. A study by Zou and colleagues determined the associations between serum visfatin levels and psoriasis [170]. This meta-analysis showed that patients with psoriasis had significantly higher levels of visfatin than controls. Furthermore, correlations showed that visfatin levels in patients were positively correlated with the PASI score. Interestingly, visfatin was found to stimulate TNF-α-induced chemokine secretion in human keratinocytes. As a result, this adipokine may contribute to the pathogenesis or exacerbation of psoriasis [171]. Additionally, Hau and colleagues, in an in vitro study, demonstrated that visfatin enhances the secretion of antimicrobial peptides in TNF-α-stimulated human keratinocytes [172].

### 4.2. Resistin

Resistin (resistance to insulin) is a hormone secreted by the adipose tissue. It was discovered in 2001 in murine adipocytes [173]. Resistin is an 11 kDa cysteine-rich polypeptide, which contains five intramolecular disulfide bonds and multiple β-turns [174]. The family of resistin-like molecules (RELM) consists of two RELM proteins in humans (RELMβ and resistin) and four RELM proteins in mice (RELMα/FIZZ1, RELMβ/FIZZ2, RELMγ, resistin) [175]. Mouse Resistin is involved in type 2 diabetes and is expressed mostly in white adipose tissue. On the contrary, the human variant is predominantly expressed in lymphatic tissue and bone marrow-derived cells, mostly in leukocytes and monocytes, and is upregulated during differentiation into macrophages [176]. The lung and heart are minor sources of resistin, and it might be involved in the remodeling of these organs after injury [177]. Resistin acts through autocrine, paracrine, and endocrine mechanisms and affects a wide variety of cell and tissue types [178]. Circulatory resistin is associated with pro-inflammatory cytokines, such as TNF-α and IL-6. The signaling activity of this protein has been found in various cell types, including macrophages, vascular cells, and peripheral blood mononuclear cells (PBMCs), among others. Nevertheless, PBMCs are believed to have the greatest influence on serum resistin levels. It is worth noting that recent studies revealed that human resistin can be also expressed in sebaceous glands and keratinocytes [179].

Moreover, resistin expression can be increased by pro-inflammatory mediators, such as TNF-α, LPS, IL-1β, and IL-6 in PBMCs [180,181,182]. Human resistin in monocytes or macrophages induces the expression of IL-12, TNF-α, and IL-6 through the NF-κB-mediated pathway [183,184]. Furthermore, resistin also induces MCP-1 secretion [185]. The overexpression of resistin can be inhibited by anti-inflammatory regiments, such as rosiglitazone or aspirin, which antagonize NF-κB [180]. In contrast, Fasshauer et al. reported that resistin mRNA expression was suppressed by TNF-α in 3T3-L1 adipocytes [186]. According to the current evidence, resistin signals through various receptors, including G-protein-coupled receptors (GPCRs), Toll-like receptor 4 (TLR4), receptor tyrosine kinase-like orphan receptor 1 (ROR1), and CAP1, an isoform of decorin (DDCN) [179].

Additionally, resistin has an immunomodulatory role. It may act as a pro-inflammatory cytokine, increase the expression of pro-inflammatory cytokines, or activate immune cells. Resistin is associated with several inflammatory, infectious, autoimmune, and neoplastic diseases [185]. Bokarewa et al. revealed that resistin introduced intraarticularly into healthy mouse joints caused arthritis and appeared in 80% of joints with injected resistin. Moreover, the authors demonstrated that patients with RA present an accumulation of resistin in synovial fluid, which is associated with the intensity of inflammation [187].

Interestingly, recent studies have started to investigate the role of resistin in the pathogenesis of psoriasis. To begin with, Johnston et al. reported that resistin is positively correlated with the severity of psoriasis [188]. Secondly, patients with psoriasis present increased levels of plasma resistin [122]. A study by Gisondi et al. confirmed that infliximab treatment reduced the serum level of resistin [189]. This finding was further confirmed by Corbetta and colleagues, who showed that treatment with the oral retinoid acitretin also caused a reduction in the resistin level [190]. Boehncke et al. noticed a statistically significant correlation between resistin serum levels and PASI score [191]. Interestingly, resistin may lead to the expansion of Tregs populations when CD4+ cells are co-cultured with dendritic cells. Moreover, resistin might inhibit the expression of IL-6, IL-12p40, and IL-23p19 through the modulation of IRF-1 in dendritic cells [192]. Since the inhibition of these cytokines might alleviate the development of psoriasis, the precise role of resistin in psoriasis is yet to be discovered.

### 4.3. Chemerin

Chemerin is an adipocyte-secreted adipokine and chemoattractant protein for dendritic cells and macrophages. Chemerin works in autocrine, paracrine, and even endocrine models of action [193]. Chemerin has a broad range of functions and takes part in adipogenesis, glucose homeostasis, and inflammation [194]. Furthermore, chemerin is believed to be a significant marker in tumorigenesis. Chemerin expression is increased in a number of inflammatory and metabolic diseases, such as metabolic syndrome, diabetes, obesity, and psoriasis [193]. The *RARRES2* gene was identified in 1997 as a new retinoid-responsive gene, upregulated in psoriatic skin after the application of tazarotene. The encoded protein of the *RARRES2* gene was identified six years later. It is also known as tazarotene-induced gene 2 (TIG2) or retinoic acid receptor responder 2 (RARRES2) [194,195].

The highest concentration of chemerin is found in the white adipose tissue, liver, and placenta. Chemerin is produced to a lesser extent by the kidneys, lungs, heart, pancreas, skeletal muscle, and brown adipose tissue. It is synthesized as preprochemerin and processed by various members of the fibrinolytic, coagulation, and inflammatory pathways. Chemerin binds to several receptors, including chemokine-like receptor 1 (CMKLR1), known as Chemerin receptor 1, G-protein coupled receptor 1 (GPR1) also named chemerin receptor 2, and C-C chemokine receptor-like 2 (CCRL2) [196]. Various signaling pathways are considered to be stimulated, such as the AMPK, MAPK, and Akt pathways [197]. For instance, Wittamer et al. observed that CMKLR1 activation results in the promotion of p42–p44 MAP kinases and suppressed cAMP accumulation [198]. In contrast, CCRL2 downstream has not yet been evaluated [199].

Recent studies suggest that chemerin plays a role in metabolic disorders. Liang et al. suggested that chemerin levels in adipose tissue and peripheral blood were elevated in women with gestational diabetes [200]. Moreover observed that patients with type 2 diabetes present higher chemerin plasma levels compared to control groups [201], whereas Bobbert et al. reported that chemerin might be a predictor of the disease [202]. In this context, it is interesting to note that Bozaoglu et al. observed that chemerin positively correlates with waist-to-hip ratio, body mass index, glucose levels, blood pressure, and circulating triglycerides [203]. Furthermore, chemerin levels were found to be positively correlated with markers of inflammation, such as CRP, IL-6, and TNF-α [204]. Interestingly, chemerin was reported to play a role in regulating adipocyte differentiation and local/autocrine actions through the CMKLR1 receptor in adipocytes [205]. Chemerin also remains an important protein in cardiovascular diseases. Kaur et al. reported that angiogenesis in human endothelial cells was induced by chemerin. Moreover, significant angiogenic pathways, MAPKs and PI3K/Akt, were activated by chemerin [206]. Xiaotao and colleagues noticed that elevated chemerin levels might indicate the severity of atherosclerosis [207]. In addition, serum chemerin levels are higher in patients with atrial fibrillation [208].

A few studies have investigated the role of chemerin in psoriasis. To begin with, a meta-analysis by Bai and colleagues revealed that serum chemerin was elevated in patients with psoriasis [209]. Secondly, the elevated level was found to decrease after the treatment with infliximab [189]. Interestingly, chemerin might have a pivotal role in the pathogenesis of psoriasis. Skrzeczyńska-Moncznik et al. demonstrated that CMKLR1+ cells migrate towards psoriatic skin [210]. This finding was supported by a separate study by Albanesi et al. [211]. Plasmacytoid dendritic cells (pDC) express CMKLR1 receptor [212], which indicates that chemerin is involved in the early stages of the disease, as it promotes the migration of cells that actively take part in the development of psoriasis. Furthermore, chemerin was found to activate NF-κB and stimulate the expression of the pro-inflammatory cytokines IL-8, IL-6, and TNF-α, which are crucial in psoriasis pathogenesis. In addition, it promoted the expression of keratin 16, of which elevated expression is observed in psoriasis and has been associated with keratinocyte proliferation [213,214]. Chemerin levels are elevated in psoriatic skin compared to healthy tissue and atopic dermatitis. Plasmacytoid dendritic cells abundantly infiltrate psoriatic skin [211], which play a significant role in the initiation of the inflammatory reactions correlated with psoriasis.

### 4.4. Irisin

Irisin is a recently discovered peptide secreted by muscle and adipose tissues [215,216,217]. Irisin promotes the browning of white adipose tissue and might impact glucose metabolism by regulating GLUT4 expression and glycolysis [218]. Furthermore, Dong and colleagues demonstrated that irisin might have anti-inflammatory properties by downregulating a marker of M1 polarization (CD86) and promoting the expression of CD206 and CD163, which belong to the M2 variant [219]. In line with this finding, Mazur-Bialy and colleagues showed that the pre-treatment of LPS-induced macrophages with irisin decreased pro-inflammatory cytokines (MCP-1, IL-6, TNFα, IL-1β) [220]. Very few studies investigated the role of irisin in psoriasis. Ambrogio et al. showed that the difference in serum irisin levels between psoriatic patients and healthy controls was marginal. The authors also found a negative correlation between irisin levels and the PASI score, but it was subsequently lost in the multivariate analysis [221]. Moreover, in a study by Baran et al., the authors found that irisin serum level was elevated compared to healthy patients, but the result was not significant. Additionally, the level of irisin did not change significantly after the psoriasis treatment. Nevertheless, the authors found positive correlations between irisin and inflammatory markers. Therefore, irisin may be a marker of inflammation in psoriasis [222].

### 4.5. C1q/tumor Necrosis Factor-Related Protein 3

C1q/tumor necrosis factor-related protein 3 (CTRP3) was first described in 2001 and named CORS26, which stands for collagenous repeat-containing sequence 26 kDa protein [223]. Subsequently, in 2004, Wong and colleagues renamed CORS26 to CTRP3 and classified it as a member of a family of adiponectin paralogs [224]. It lowers blood glucose levels, and a reduced concentration of CTRP3 is found in patients with diabetes [225,226]. Interestingly, CTRP3 was found to promote the secretion of leptin, adiponectin, and visfatin in 3T3-L1 adipocytes [227]. Furthermore, several studies on different cell types and with animal models demonstrated that CTRP3 may promote AMPK phosphorylation and SIRT1 enhancement [228,229,230,231,232]. To date, little is known about the role of CTRP3 in the development of psoriasis. AdipoR2 has been recently identified as one of the receptors for CTRP3 in the chondrocyte cell line [233]. CTRP3–AdipoR2 interaction was subsequently found to inhibit Th17 cell differentiation [234]. Xue et al. showed that CTRP3 levels were lower in psoriasis patients compared with healthy controls. In addition, the authors showed that CTRP3 can suppress keratinocyte inflammation through the inhibition of STAT3 phosphorylation [235], which indicates that CTRP3 might have a protective effect on the development of psoriasis. A summary of selected adipokines and relevant mechanisms in the pathogenesis of psoriasis is presented in Table 1.

## 5. Conclusions

Psoriasis is a widespread disease caused by numerous factors, such as stress, infections, or smoking. A genetic component may also contribute to the development of the disease. Within psoriasis, several processes with multiple mediators are activated, and adipokines might play a significant role. These hormones have different effects on immune responses in the skin. Adiponectin levels are reduced in psoriasis patients, and this is thought to affect the production of IL-23 and IL-17. Leptin is associated with the promotion of pro-inflammatory cytokines and psoriasis-related cells, such as Th17. Resistin and visfatin also exhibit pro-inflammatory actions and are elevated in psoriasis patients. Chemerin might be associated with the early stages of psoriasis. Further research is required to evaluate the role of irisin in inflammatory disorders. In summary, adipokines may have pro- and anti-inflammatory functions. In addition, many novel adipokines have been recently identified, such as follistatin-like 1 (FSTL1), wingless-type inducible signaling pathway protein 1 (WISP1), or Asprosin, among others. Recent evidence suggests they play a role in the development of metabolic diseases [236], but further studies need to investigate their immunomodulatory properties and impact on psoriasis. The increasing number of discovered adipokines determines the development of an interesting research field.

## Figures and Tables

**Table 1:** Summary of selected adipokines and mechanisms related to the pathogenesis of psoriasis.

Adipokine	Expression in Psoriasis	Psoriasis-Related Immunomodulatory Effects	Reference
Adiponectin	Decreased	Negative correlation between adiponectin and IL-23 gene expression in patients with high LDL Adiponectin deficiency promotes IL-23p19 and IL-17 Adiponectin suppresses IL-17 production in IL-23-stimulated dermal γδ-T cells Intraperitoneal injection of adiponectin resulted in inhibition of IL-17 production in adiponectin-deficient mice	[122,123,126,127]
Leptin	Elevated	Leptin Promotes Th17 differentiation Leptin neutralization promotes Treg proliferation	[26,153,155,160]
Visfatin	Elevated	Visfatin enhances inflammatory responses in keratinocytes induced by TNF-α	[170,171,172]
Resistin	Elevated	Resistin may suppress IL-23p19 in dendritic cells	[122,188,192]
Chemerin	Elevated	Chemerin might promote migration of dendritic cells, which actively take part in psoriasis pathogenesis Chemerin promotes secretion of pro-inflammatory cytokines in keratinocytes	[209,210,212,213]

## Data Availability

Not applicable.
